# Integration of the systemic inflammatory response index with pulse pressure enhances prognostication of cardiovascular mortality in the general population of the United States: insights from the NHANES database

**DOI:** 10.3389/fcvm.2024.1439239

**Published:** 2024-11-18

**Authors:** Jie An, Zikan Zhong, Bingquan Xiong, Dandan Yang, Youquan Li, Ya Luo, Hao Li, Yang Jiao, Genqing Zhou, Min Xu, Shaowen Liu, Jie Li

**Affiliations:** ^1^ Department of Cardiology, The Third Affiliated Hospital of Zunyi Medical University (The First People’s Hospital of Zunyi), Guizhou, China; ^2^ Department of Cardiology, Shanghai General Hospital, Shanghai Jiao Tong University School of Medicine, Shanghai, China; ^3^ Department of Cardiology, The Second Affiliated Hospital of Chongqing Medical University, Chongqing, China; ^4^ Department of Pediatrics, The Third Affiliated Hospital of Zunyi Medical University (The First People’s Hospital of Zunyi), Guizhou, China; ^5^ Department of Interventional Treatment Centers, The Third Affiliated Hospital of Zunyi Medical University (The First People’s Hospital of Zunyi), Guizhou, China

**Keywords:** systemic inflammatory response index, pulse pressure, cardiovascular mortality, prognostication, nomogram

## Abstract

**Background:**

The prognostic significance of utilizing both the systemic inflammatory response index (SIRI) and pulse pressure (PP) collectively in assessing cardiovascular mortality (CVM) across populations remains to be elucidated.

**Methods:**

Multivariate Cox proportional hazards analysis investigated the SIRI, PP, and CVM association. Receiver operating characteristic (ROC) curves evaluated the predictive performance of the combined SIRI and PP for CVM in the broader demographic. Subsequently, the area under the ROC curve (AUC) was compared using the *Z*-test, and a novel nomogram was developed to assess its accuracy in predicting CVM. Restricted cubic spline (RCS) was used to evaluate the association between SIRI and PP.

**Results:**

The study involved 19,086 NHANES database individuals, with 9,531 males (49.94%). During the follow-up period, 456 CVM instances (2.39%) occurred. Multivariate Cox proportional hazards analysis revealed both the SIRI [adjusted hazard ratio (HR) 1.16, *P* < 0.001] and PP (HR = 1.01, *P* = 0.004) as independent CVM predictors. A 0.1-unit SIRI increase and 10 mmHg PP escalation correlated with 2% (adjusted HR = 1.02, *P* < 0.001) and 7% (adjusted HR = 1.07, *P* = 0.004) CVM enhancements, respectively. The combined SIRI and PP area under the curve was 0.77, ranging from 0.77 to 0.79 in female cohorts, non-smokers, and non-pathological contexts. High SIRI and PP, either high SIRI or PP, were associated with 3 and 2 times the CVM risk compared to low SIRI and PP. Adding the SIRI and PP to general risk factors improved CVM predictive efficacy (Z = 4.17, *P* < 0.001). The novel nomogram's concordance index was 0.90, indicating excellent discrimination. The predicted probabilities’ calibration plot aligned with actual CVM rates at 1, 5, and 10 years. RCS showed an S-shaped relationship between SIRI and PP.

**Conclusions:**

Integrating the SIRI with PP demonstrates substantial predictive efficacy for CVM within the broader United States community, notably in female cohorts, non-smokers, and non-pathological contexts.

## Background

Cardiovascular disease (CVD) poses a significant global health challenge, accounting for approximately 22.9% of all deaths (1). The rising incidence of CVD necessitates effective diagnostic and prognostic tools. Chronic inflammation is central to CVD pathogenesis, contributing to endothelial dysfunction, atherosclerosis, and increased cardiovascular mortality (CVM) (2–5). Among inflammatory markers, the systemic inflammatory response index (SIRI) has emerged as a notable predictor of adverse cardiovascular outcomes. Elevated SIRI levels correlate with worse cardiovascular outcomes, indicating its potential as a diagnostic marker of inflammatory status and its implications for cardiovascular health (2, 6–9).

Simultaneously, pulse pressure (PP) often occupies a subordinate position in clinical assessments compared to its systolic and diastolic counterparts. Nonetheless, it is a pivotal indicator of arterial stiffness and cardiovascular risk. Beyond its portrayal of arterial blood flow culpability, PP provides intricate insights into the dynamic interplay among cardiac output, vascular compliance, and peripheral resistance. Extensive literature has elucidated the close association of PP with the risk of stroke, myocardial infarction, heart failure, diabetes, and CVM (10–14). However, investigations into the relationship between PP and CVM in elderly patient cohorts remain limited, yielding disparate findings (15). Thus, PP serves not only as a measure of blood pressure but also as an indicator of underlying vascular pathology.

The interplay between SIRI and PP is compelling, as both metrics reflect distinct yet interrelated aspects of cardiovascular health. SIRI encapsulates systemic inflammation, while PP provides insights into hemodynamic changes and vascular compliance. Chronic inflammation may induce vascular remodeling and stiffening (16), potentially linking elevated SIRI with increased PP in at-risk populations. Additionally, inflammation disrupts blood pressure regulation, contributing to elevations in both systolic and diastolic pressures, thereby influencing PP.

Despite the established prognostic value of SIRI and PP, research exploring their combined predictive power for CVM is limited. Utilizing both SIRI and PP as complementary indicators could enhance risk stratification, aiding clinicians in identifying individuals at heightened risk for cardiovascular events. Ultimately, in this context, we propose a novel clinical prediction model that incorporates both PP and SIRI. Prior nomograms have demonstrated an AUC ranging from 0.70 to 0.84 in predicting all-cause mortality and CVM. We aim to investigate a novel nomogram that integrates SIRI and PP to ascertain whether it offers enhanced diagnostic value in forecasting CVM, potentially providing clinicians with a more robust tool for identifying individuals at increased risk (17–19).

### Study population

NHANES, an acronym for the National Health and Nutrition Examination Survey in the US, integrates an extensive range of data collection methodologies. These encompass questionnaire surveys, physical examinations, analysis of biological specimens, and nutritional assessments. This survey serves as a pivotal tool for gaining insights into the health status of the American populace, assessing the efficacy of public health policies, and informing strategic initiatives in clinical practice. Our study utilized data spanning from 2007 to 2016, which can be accessed through the official website. All data are integrated using unique respondent sequence number (SEQN) identifiers, with follow-up outcomes recorded up to December 31, 2019. Following the exclusion of missing values and outliers, a total of 19,086 individuals were included in the study cohort. A detailed flowchart depicting participant selection is provided in Supplementary Figure 1.

### Data collection and definitions

Data were collected by matching unique SEQN identifiers and including information on age, gender, race, educational attainment, marital status, smoking status, drinking habits, income-to-poverty ratio (PIR ≥ 2.5, <2.5), diabetes mellitus (DM), congestive heart failure (CHF), cancer, coronary heart disease (CHD), hypertension, stroke, chronic kidney disease (CKD), body mass index (BMI), daily energy intake, sleep time, systemic inflammatory response index (SIRI), systemic immune-inﬂammation index (SII), systemic immune-inﬂammation response index (SIIRI), systolic blood pressure (SBP), diastolic blood pressure (DBP), mean artery pressure (MAP), pulse pressure (PP), and cardiovascular mortality. Data containing anomalies or missing values is excluded. Drinking habits are operationally defined as the consumption of a minimum of 12 alcoholic beverages within the preceding year. Smoking status is operationally defined as having a cumulative history of tobacco consumption amounting to a minimum of 100 cigarettes for one's lifetime. Body mass index (BMI) is computed by dividing an individual's weight (measured in kilograms) by the square of their height (measured in meters) (20). SBP and DBP were assessed on at least three separate occasions, and the mean of these measurements was determined by at least two researchers. The calculation formulas for SIRI, SII, and SIIRI are as follows (21):

$$\mathrm{SII}\;=\frac{\;\mathrm{platelet}\;\mathrm{count}\;x\;\mathrm{neutrophil}\;\mathrm{count}\;}{\;\mathrm{lymphocyte}\;\mathrm{count}\;}$$

$$\mathrm{SIRI}\;=\frac{\;\mathrm{monocyte}\;\mathrm{count}\;x\;\mathrm{neutrophil}\;\mathrm{count}\;}{\;\mathrm{lymphocyte}\;\mathrm{count}\;}$$

$$\mathrm{SIIRI}\;=\frac{\;\mathrm{platelet}\;\mathrm{count}\;x\;\mathrm{monocyte}\;\mathrm{count}\;x\;\mathrm{neutrophil}\;\mathrm{count}\;}{\;\mathrm{lymphocyte}\;\mathrm{count}\;}$$

### Endpoints

The study's endpoint is CVM, which signifies death from disorders and conditions affecting the heart and blood vessels. This includes fatal occurrences like heart attacks, strokes, and other cardiovascular diseases.

### Statistical analysis

Continuous variables that adhere to a normal distribution are presented as mean ± standard deviation. Analysis entails either an independent two-sample *t*-test or an analysis of variance. Conversely, median and interquartile ranges are utilized for non-normally distributed data, and analysis is conducted using non-parametric tests. Categorical data are represented as percentages and analyzed employing the chi-square test. Univariate and multivariate Cox proportional hazards regression analyses were employed to examine the risk factors linked with CVM. The area under the receiver operating characteristic (ROC) curve (AUC) served to evaluate the predictive capacity for mortality. Cut-off values (i.e., the optimal threshold corresponding to the maximum Youden index) were then calculated for SIRI and PP. Participants were categorized into high SIRI and low SIRI, high PP and low PP, low SIRI and PP, either high SIRI or PP, and both high SIRI and PP based on these cut-off values. A sensitivity analysis was conducted to explore the robust predictive value of SIRI combined with PP for cardiovascular mortality. This was done in various risk factor models, excluding individuals with a follow-up period of less than 3 years, and in populations where missing SIRI and PP values were imputed. Kaplan-Meier curves were plotted accordingly and the differences between groups were compared using the log-rank test. Calculate the AUC for the SIRI and PP combined to assess their predictive value for CVM, as well as in subgroup analyses for different populations. The ROC curve AUC was compared using the *Z*-test. RCS was utilized to investigate the nonlinear associations between continuous SIRI and PP. The determination of the number and placement of knots in the RCS was based on the Akaike information criterion (AIC), which helps to find a balance between achieving a good fit and avoiding overfitting (22). By jointly constructing a Nomogram risk prediction model based on multiple independent risk factors, we utilize the C-index to assess discrimination. The C-index is evaluated by conducting 1,000 bootstrap samples to calculate the index, with values ranging between 0.5 and 1. A value of 0.5 indicates complete inconsistency, suggesting that the model lacks predictive power, while a value of 1 signifies perfect consistency, indicating that the model's predictions align entirely with the actual outcomes (23). Additionally, we utilized calibration curves to assess the concordance between the Nomogram's predictions of CVM at 1, 5, and 10 years and the observed mortality rates. The NHANES population was divided into a training set and a validation set to enhance the robustness of model validation (24). All statistical analyses were conducted utilizing SPSS 26, GraphPad Prism 8.0, and R 4.3.1. Two-sided *P*-values < 0.05 indicate significance.

## Results

### Baseline data for study participants

Out of 50,588 data points, 28,675 had missing or anomalous values, and 2,827 were missing SIRI and PP. Finally, 19,086 participants were included in the analysis, with 456 deaths (2.39%). The distribution included individuals age (48.9 ± 17.5 years), males (49.94%), Non-Hispanic White ethnicity (44.86%), individuals with a college degree or above (53.83%), widowed/divorced/separated individuals (21.43%), smokers (44.96%), individuals reporting drinking (72.82%), and so on. Detailed information can be found in Table 1. The SIRI and PP did not follow a normal distribution, with median and interquartile range values for SIRI being 1.02 (0.69–1.50) and for PP 50 (41–61), respectively. Based on the cutoff values of SIRI (1.55) and PP (60 mmHg), the population was divided into three groups (low SIRI and PP, either high SIRI or PP, and both high SIRI and PP). In the high SIRI and high PP groups, there were higher proportions of individuals age, smokers, individuals with PIR <2.5, and higher rates of diseases (DM, CHF, CHD, stroke, cancer, CKD, hypertension). Additionally, these individuals exhibited higher BMI and higher SBP. High SIRI and high PP, either high SIRI or high PP, and low SIRI and low PP groups had CVM rates of 9.38%, 3.61%, and 0.66%, respectively. The intergroup differences were statistically significant (*P* < 0.001), as shown in the Table 2. In the Supplementary Table 1, it was observed that the rates of smoking, drinking, DM, CHF, CKD, and CVM were lower in females when grouped by gender.

**Table 1:** General baseline characteristics of the included population.

Characteristic	Total (*n* = 19,086)	Alive (*n* = 18,630)	CVM (*n* = 456)	*p* value
Age (years)	48.9 ± 17.5	48.4 ± 17.3	70.7 ± 11.8	<0.001
Male, *n* (%)	9,531 (49.94)	9,248 (49.64)	283 (62.06)	<0.001
Race, *n* (%)				
Mexican American	2,871 (15.04)	2,837 (15.23)	34 (7.46)	<0.001
Other Hispanic	1,965 (10.30)	1,938 (10.40)	27 (5.92)	
Non-Hispanic White	8,563 (44.86)	8,273 (44.41)	290 (63.60)	
Non-Hispanic Black	3,783 (19.82)	3,689 (19.80)	94 (20.61)	
Other Race—including Multi-Racial	1,904 (9.98)	1,893 (10.16)	11 (2.41)	
Educational level, *n* (%)				
Less than high school	4,486 (23.50)	4,322 (23.20)	164 (35.96)	<0.001
High school or equivalent	4,327 (22.67)	4,207 (22.58)	120 (26.32)	
College or above	10,273 (53.83)	10,101 (54.22)	172 (37.72)	
Marital status, *n* (%)				
Married or cohabiting	11,498 (60.24)	11,273 (60.51)	225 (49.34)	<0.001
Never married	3,498 (18.33)	3,457 (18.56)	41 (9.00)	
Widowed/divorced/separated	4,090 (21.43)	3,900 (20.93)	190 (41.66)	
Smoke status, *n* (%)	8,582 (44.96)	8,333 (44.73)	249 (54.61)	<0.001
Drinking, *n* (%)	13,899 (72.82)	13,592 (72.96)	307 (67.32)	0.008
Income-to-poverty ratio, *n* (%)				
<2.5	10,691 (56.01)	10,378 (55.71)	313 (68.64)	<0.001
≥2.5	8,395 (43.99)	8,252 (44.29)	143 (31.36)	
Diabetes mellitus, *n* (%)	2,437 (12.77)	2,286 (12.27)	151 (33.11)	<0.001
Congestive heart failure, *n* (%)	556 (2.91)	466 (2.50)	90 (19.74)	<0.001
Coronary heart disease, *n* (%)	738 (3.87)	639 (3.43)	99 (21.71)	<0.001
Stroke, *n* (%)	677 (3.55)	614 (3.30)	63 (13.82)	<0.001
Cancer, *n* (%)	1,789 (9.37)	1,704 (9.15)	85 (18.64)	<0.001
Chronic kidney disease, *n* (%)	551 (2.89)	513 (2.76)	38 (8.33)	<0.001
Hypertension, *n* (%)	6,646 (34.82)	6,332 (33.99)	314 (68.86)	<0.001
Body mass index (km/m^2^)	29.09 ± 6.78	29.07 ± 6.68	29.64 ± 7.08	<0.001
Daily energy intake (kcal/day)	2,036 ± 783	2,042 ± 784	1,761 ± 673	0.08
Sleep time (h)	7 (6–8)	7 (6–8)	7 (6–8)	0.039
SIRI	1.02 (0.69–1.50)	1.00 (0.69–1.48)	1.54 (0.97–2.26)	<0.001
SII	457.76 (328.21–642.34)	455.69 (327.13–638.41)	553.04 (388.23–785.00)	<0.001
SIIRI	239.33 (154.53–371.67)	238.00 (153.73–368.33)	330.91 (194.12–512.73)	<0.001
Systolic blood pressure (mmHg)	121 (111–133)	120 (111–132)	132 (119–148)	<0.001
Diastolic blood pressure (mmHg)	70 (63–77)	71 (63–77)	65 (56–73)	<0.001
Mean artery pressure (mmHg)	87 (80–94)	87 (80–94)	88 (80–96)	0.257
Pulse pressure (mmHg)	50 (41–61)	50 (41–61)	66 (53–81)	<0.001

Data are presented as mean (standard deviation) or median (interquartile) or *n* (%), CVM cardiovascular mortality; BMI, body mass index; SIIRI, systemic immune-inflammation response index; SII, systemic immune-inflammation index; SIRI, systemic inflammation response index.

**Table 2:** Baseline characteristics based on SIRI and PP cutoff value grouping.

Characteristic	Low SIRI and low PP	Either high SIRI or high PP	High SIRI and high PP	*p* value
	(*n* = 10,818)	(*n* = 6,765)	(*n* = 1,503)	
Age (years)	43.39 ± 14.84	54.24 ± 18.04	63.64 ± 17.32	<0.001
Male, *n* (%)	5,086 (47.01)	3,517 (51.99)	928 (61.74)	<0.001
Race, *n* (%)				<0.001
Mexican American	1,671 (15.45)	1,028 (15.20)	172 (11.44)	
Other Hispanic	1,153 (10.66)	680 (10.05)	132 (8.78)	
Non-Hispanic White	4,521 (41.79)	3,118 (46.09)	924 (61.48)	
Non-Hispanic Black	2,188 (20.22)	1,400 (20.70)	195 (12.98)	
Other Race—including Multi-Racial	1,285 (11.88)	539 (7.96)	80 (5.32)	
Educational level, *n* (%)				<0.001
Less than high school	2,217 (20.49)	1,871 (27.66)	398 (26.48)	
High school or equivalent	2,307 (21.33)	1,624 (24.01)	396 (26.35)	
College or above	6,294 (58.18)	3,270 (48.33)	709 (47.17)	
Marital status, *n* (%)				<0.001
Married or cohabiting	6,746 (62.36)	3,900 (57.65)	852 (56.69)	
Never married	2,279 (21.07)	1,055 (15.59)	164 (10.91)	
Widowed/divorced/separated	1,793 (16.57)	1,810 (26.76)	487 (32.40)	
Smoke status, *n* (%)	4,392 (40.60)	3,324 (49.14)	866 (57.62)	<0.001
Drinking, *n* (%)	8,036 (74.28)	4,772 (70.54)	1,091 (72.59)	<0.001
Income-to-poverty ratio, *n* (%)				<0.001
<2.5	5,792 (53.54)	4,021 (59.44)	878 (58.42)	
≥2.5	5,026 (46.46)	2,744 (40.56)	625 (41.58)	
Diabetes mellitus, *n* (%)	877 (8.11)	1,187 (17.55)	373 (24.82)	<0.001
Congestive heart failure, *n* (%)	161 (1.49)	265 (3.92)	130 (8.65)	<0.001
Coronary heart disease, *n* (%)	192 (1.77)	387 (5.72)	159 (10.58)	<0.001
Stroke, *n* (%)	215 (1.99)	324 (4.79)	138 (9.18)	<0.001
Cancer, *n* (%)	693 (6.41)	778 (11.50)	318 (21.16)	<0.001
Chronic kidney disease, *n* (%)	203 (1.88)	244 (3.61)	104 (6.92)	<0.001
Hypertension, *n* (%)	2,628 (24.29)	3,093 (45.72)	925 (61.54)	<0.001
Body mass index (km/m^2^)	28.62 ± 6.60	29.64 ± 6.90	29.93 ± 7.33	<0.001
Daily energy intake (kcal/day)	2,082 ± 793	1,988 ± 772	1,921 ± 726	<0.001
Sleep time (h)	7 (6–8)	7 (6–8)	7 (6–8)	<0.001
Cardiovascular mortality, *n* (%)	71 (0.66)	244 (3.61)	141 (9.38)	<0.001

Data are presented as mean (standard deviation) or median (interquartile) or *n* (%), SIRI, systemic inflammation response index; PP, pulse pressure; BMI, body mass index.

### Correlation and predictive value of SIRI, PP, and CVM

**Figure 1:**
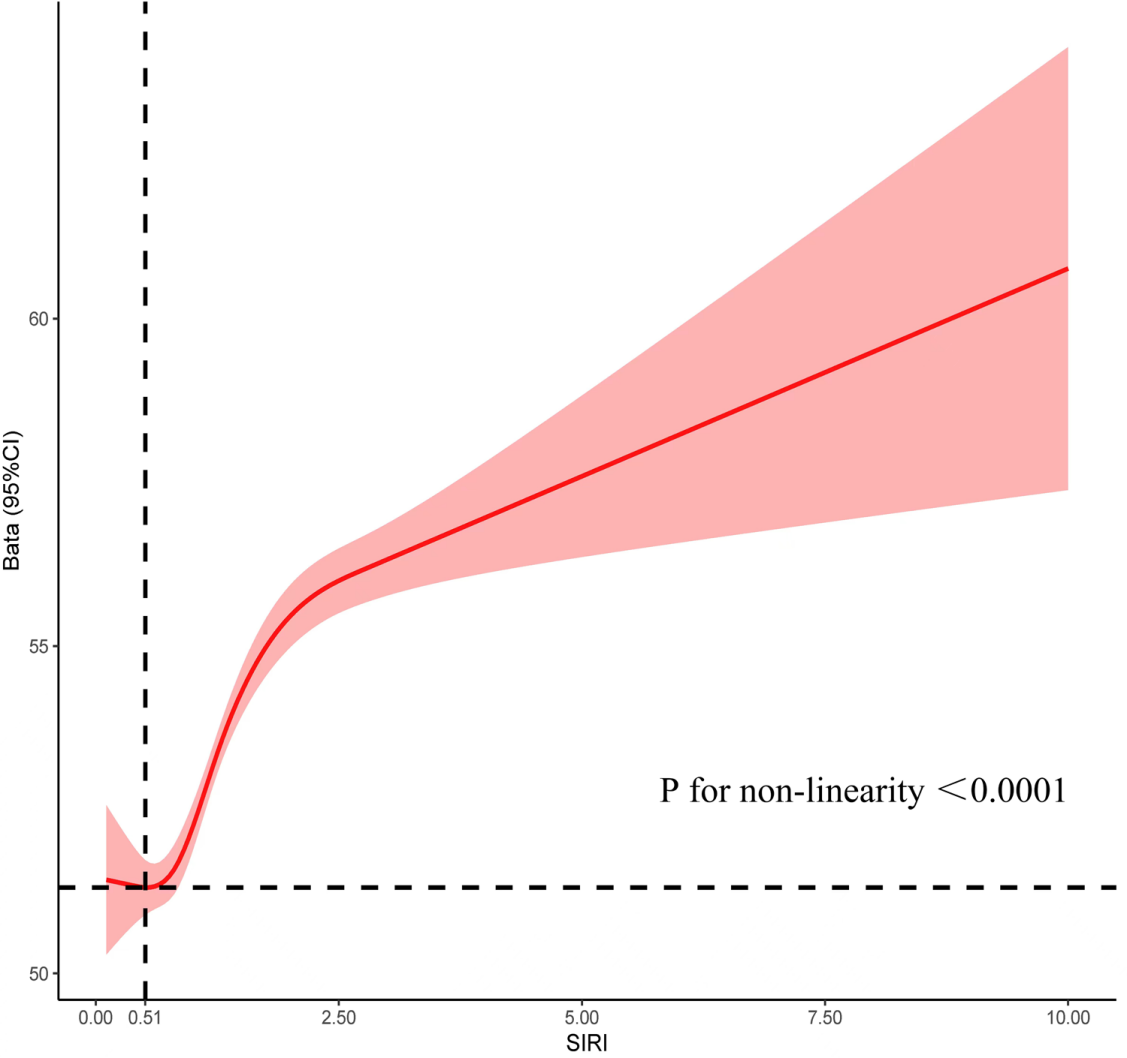
Restrictive cubic spline explores the relationship between SIRI and PP, with SIRI on the *X*-axis and PP on the *Y*-axis.

**Figure 2:**
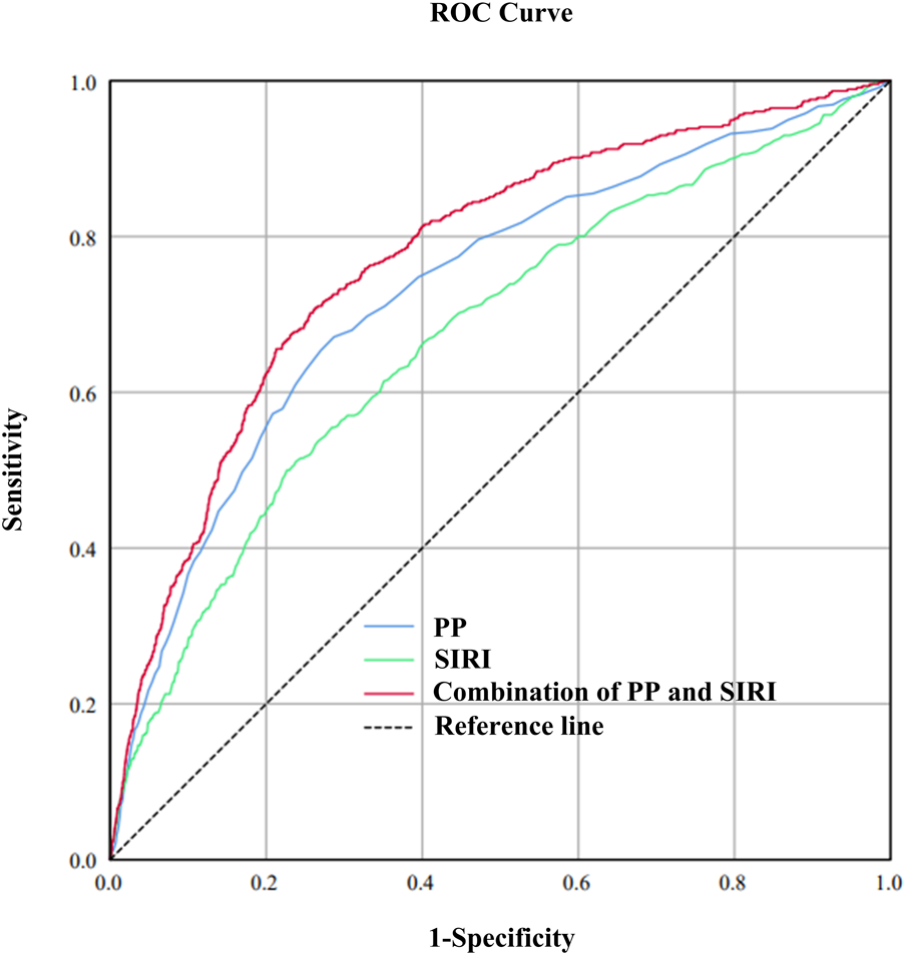
To compare the predictive value of SIRI, PP, and the combined SIRI and PP for cardiovascular mortality, the area under the receiver operating characteristic (ROC) curve (AUC) was calculated. The calculated AUC values were 0.67, 0.73, and 0.77 for SIRI, PP, and the combined SIRI and PP, respectively.

Incorporating indicators such as SIRI, SII, and SIIRI for assessing CVM, the AUC values were 0.67, 0.60, and 0.62 (Supplementary Figure 2). Incorporating indicators such as PP, SBP, DBP, and MAP for assessing CVM, the AUC values were 0.73, 0.67, 0.61, and 0.52, respectively (Supplementary Figure 3). There were significant differences observed between the alive group and the CVM group in both SIRI [1.00 (0.69–1.48) vs. 1.54 (0.97–2.26), *P* < 0.001] and PP [50 (41–61) vs. 66 (53–81), *P* < 0.001]. RCS showed an S-shaped relationship between SIRI and PP (*P* < 0.0001). When SIRI exceeded 0.51, PP sharply increased as SIRI increased, followed by a gradual rise (Figure 1). The cutoff value for SIRI was 1.55, with a sensitivity of 50.0% and a specificity of 77.4%. For PP, the cutoff value was 60 mmHg, with a sensitivity of 65.4% and a specificity of 73.0%. When combining SIRI and PP, the AUC was 0.77 (95% CI: 0.75–0.80), with a sensitivity of 70.2% and specificity of 74.4% (Figure 2). Using the *Z*-test to compare the area under the ROC curve (AUC), statistical differences were found between the combined SIRI and PP and SIRI (Z = 6.78, *P* < 0.001), as well as between the combined SIRI and PP and PP (Z = 6.86, *P* < 0.001) (Supplementary Table 2a).

### Univariate and multivariate cox regression analysis of CVM

Multivariate Cox regression analysis revealed that gender, age, race, marital status, DM, CHF, CHD, cancer, hypertension, smoke status, PIR, SIRI, and PP were independent risk factors for CVM (Table 3). The general risk factors are defined as age, gender, race, marital status, DM, CHF, CHD, cancer, hypertension, and PIR. Here are the definitions for the groups: Group 1: High SIRI and high PP; Group 2: Either high SIRI or high PP; Group 3: Low SIRI and low PP. In the unadjusted model, a 0.1-unit increase in SIRI had an HR of 1.03, and a 10 mmHg increase in PP had an HR of 1.47. When comparing Group 1 to Group 3, the HR was 18.18. For comparisons of Group 2 against Group 1, the HR was 5.97. In model 1, adjusting for age and gender, for each 0.1-unit increase in SIRI, the HR was 1.02. For every 10 mmHg increase in PP, the HR was 1.10. When comparing Group 3 with Group 1, the HR was 2.28. The comparison of Group 2 with Group 1 resulted in an HR of 3.80. In model 2, adjusting for general risk factors and PP, for each 0.1-unit increase in SIRI, the HR was 1.02. Additionally, adjusting for general risk factors and SIRI, for every 10 mmHg increase in PP, the HR was 1.07. When adjusting for general risk factors and comparing Group 3 with Group 1, the HR was 3.17. The comparison of Group 2 with Group 1 resulted in an HR of 1.99. The comparison among these three groups yielded a *P* for trend <0.001 (Table 4).

**Table 3:** Univariate and multivariate cox regression analysis.

Variables	Univariate analysis		Multivariate analysis	
	HR (95%CI)	*P* value	HR (95%CI)	*P* value
Age	1.11 (1.10–1.12)	<0.001	1.09 (1.08–1.10)	<0.001
Gender	1.66 (1.37–2.01)	<0.001	1.97 (1.56–2.48)	<0.001
Smoke status	1.48 (1.23–1.78)	<0.001	0.94 (0.77–1.16)	0.561
Drinking	1.35 (1.11–1.65)	0.002	1.02 (0.81–1.27)	0.89
PIR	1.77 (1.45–2.16)	<0.001	1.49 (1.20–1.85)	<0.001
DM	3.84 (3.16–4.66)	<0.001	1.52 (1.23–1.88)	<0.001
CHF	11.13 (8.83–14.03)	<0.001	2.67 (2.05–3.47)	<0.001
CHD	8.36 (6.69–10.45)	<0.001	1.60 (1.24–2.06)	<0.001
Stroke	5.16 (3.95–6.73)	<0.001	1.23 (0.93–1.62)	0.150
Cancer	2.46 (1.95–3.12)	<0.001	1.40 (1.09–1.79)	0.007
CKD	3.77 (2.71–5.26)	<0.001	1.31 (0.92–1.84)	0.132
Hypertension	4.48 (3.68–5.47)	<0.001	1.24 (1.00–1.53)	0.055
SIRI	1.04 (1.04–1.04)	<0.001	1.16 (1.12–1.21)	<0.001
PP	1.30 (1.27–1.34)	<0.001	1.01 (1.01–1.01)	0.004
BMI	1.01 (1.00–1.03)	0.059	1.01 (1.00–1.03)	0.358
Daily energy intake	1.00 (1.00–1.00)	<0.001	1.00 (1.00–1.00)	0.187
Sleep time	1.16 (1.09–1.24)	<0.001	1.06 (1.00–1.12)	0.59
Educational level				
Less than high school	2.11 (1.71–2.62)	<0.001	1.02 (0.80–1.30)	0.35
High school or equivalent	1.63 (1.29–2.05)		0.87 (0.68–1.10)	
College or above	Reference		Reference	
Marital status				
Married or cohabiting	Reference	<0.001	Reference	<0.001
Never married	0.60 (0.43–0.84)		2.34 (1.65–3.32)	
Widowed/divorced/separated	2.51 (2.07–3.04)		1.47 (1.19–1.81)	
Race				
Mexican American	Reference	<0.001	Reference	<0.001
Other Hispanic	1.21 (0.73–2.00)		1.20 (0.72–2.00)	
Non-Hispanic White	2.87 (2.01–4.10)		1.88 (1.29–2.74)	
Non-Hispanic Black	2.21 (1.49–3.27)		1.85 (1.23–2.77)	
Other Race—including Multi-Racial	0.59 (0.30–1.16)		0.81 (0.41–1.61)	

PIR, income-to-poverty ratio; DM, diabetes mellitus; CHF, congestive heart failure; CHD, coronary heart disease; CKD, chronic kidney disease; SIRI, systemic inflammation response index; PP, pulse pressure; BMI, body mass index.

**Table 4:** Cox proportional hazard analysis for cardiovascular mortality using SIRI and PP.

Variables	Unadjusted model		Multivariate analysis			
	HR (95%CI)	*P* value	Model 1		Model 2	
			HR (95%CI)	*P* value	HR (95%CI)	*P* value
SIRI per 0.1unit	1.03 (1.02–1.03)	<0.001	1.02 (1.01–1.02)	<0.001	1.02 (1.01–1.02)i	<0.001
PP per 10 mmHg	1.47 (1.42–1.52)	<0.001	1.10 (1.04–1.15)	<0.001	1.07 (1.02–1.13)ii	0.004
Low SIRI and low PP	Reference		Reference		Reference	
Either high SIRI or high PP	5.97 (4.58–7.77)	<0.001	2.28 (1.73–3.01)	<0.001	1.99 (1.51–2.63)iii	<0.001
High SIRI and high PP	18.18 (13.66–24.19)	<0.001	3.80 (2.79–5.18)	<0.001	3.17 (2.31–4.34)iii	<0.001
*P* for trend	<0.001		<0.001		<0.001	
Imputation of missing values						
Low SIRI and low PP	Reference		Reference		Reference	
Either high SIRI or high PP	4.97 (3.96–6.24)	<0.001	1.91 (1.50–2.42)	<0.001	1.71 (1.34–2.18)iii	<0.001
High SIRI and high PP	13.60 (10.59–17.47)	<0.001	2.83 (2.16–3.72)	<0.001	2.45 (1.86–3.22)iii	<0.001
*P* for trend	<0.001		<0.001		<0.001	
Follow-up time ≥ 3 years						
Low SIRI and low PP	Reference		Reference		Reference	
Either high SIRI or High PP	4.71 (3.34–6.64)	<0.001	2.42 (1.71–3.44)	<0.001	2.09 (1.47–2.98)iii	<0.001
High SIRI and high PP	14.86 (10.51–21.00)	<0.001	3.24 (2.25–4.68)	<0.001	2.74 (1.90–3.97)iii	<0.001
*P* for trend	<0.001		<0.001		<0.001	

Model 2i adjusting for general risk factors and PP.

Model 2ii adjusting for general risk factors and SIRI.

Model 2iii adjusting for general risk factors.

The general risk factors are defined as age, gender, race, marital status, diabetes mellitus, congestive heart failure, coronary heart disease, cancer or malignancy, hypertension, total energy, sleep time and income-to-poverty ratio. SIRI, systemic inflammation response index; PP, pulse pressure; HR, hazard ratio; CI, confidence interval.

### Sensitivity analysis and subgroup analysis

Imputation of missing values for SIRI and PP: The HR for Group 3 vs. Group 1 in the unadjusted model, Model 1, and Model 2 were 13.60, 2.83, and 2.45, respectively. For Group 2 vs. Group 1, the HRs were 4.97, 1.91, and 1.71, respectively. After excluding data with follow-up times of less than 3 years, the HR for Group 3 vs. Group 1 in the unadjusted model, Model 1, and Model 2 were 14.86, 3.24, and 2.74, respectively. For Group 2 vs. Group 1, the HRs were 4.71, 2.42, and 2.09, respectively.

### Predictive value of the ROC curve combining SIRI and PP in different populations

**Figure 3:**
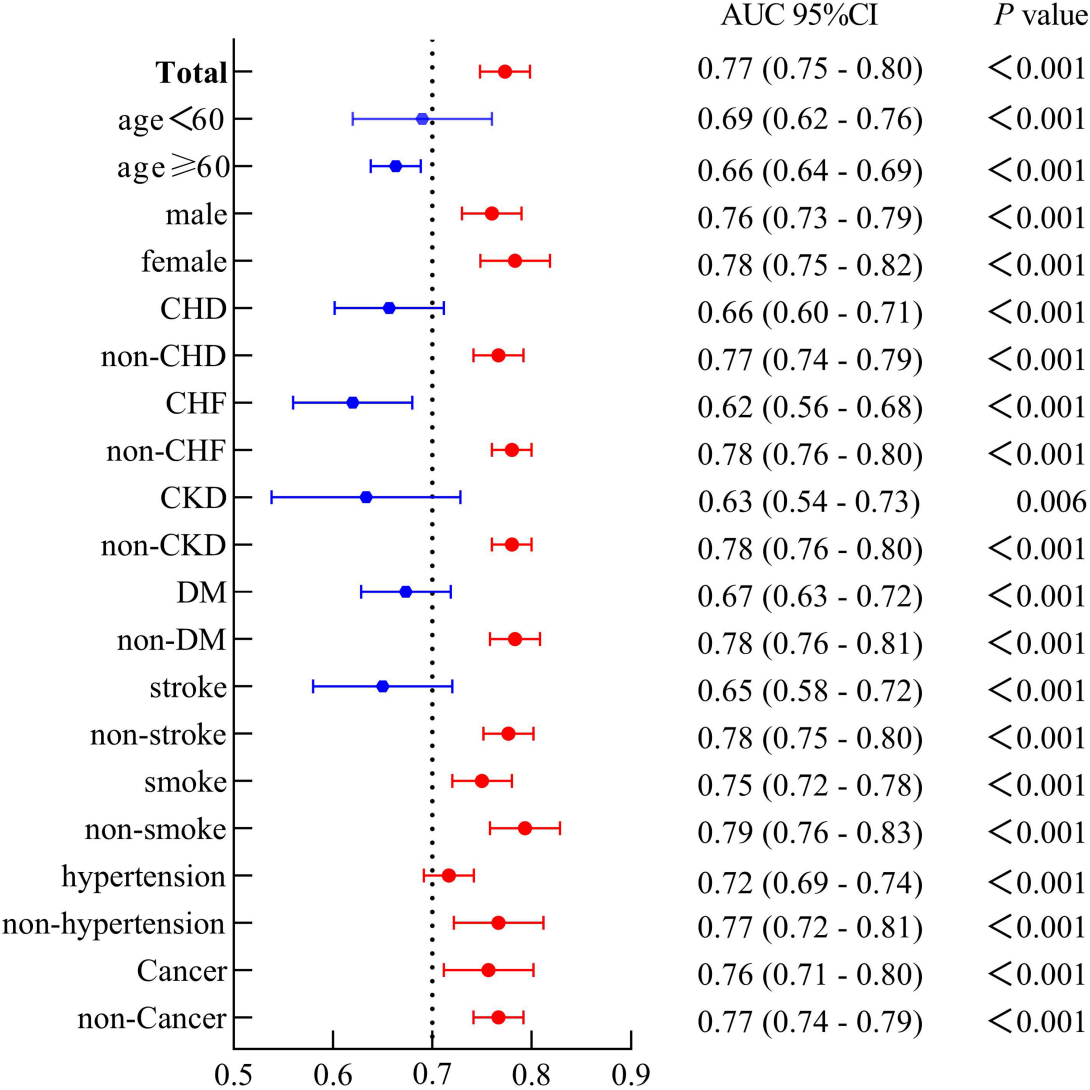
The forest plot demonstrated the combined predictive value of SIRI and PP among different subgroups.

As depicted in forest plot Figure 3, SIRI combined with PP demonstrated uniform predictive value for CVM across all subgroups. The overall area under the curve (AUC) was 0.77, with sensitivity of 70.2% and specificity of 74.4%. The highest predictive value was observed in non-smokers (AUC: 0.79), while the predictive value was high and stable in female and non-pathological contexts (AUC: 0.77–0.78).

### Kaplan-Meier survival curve

**Figure 4:**
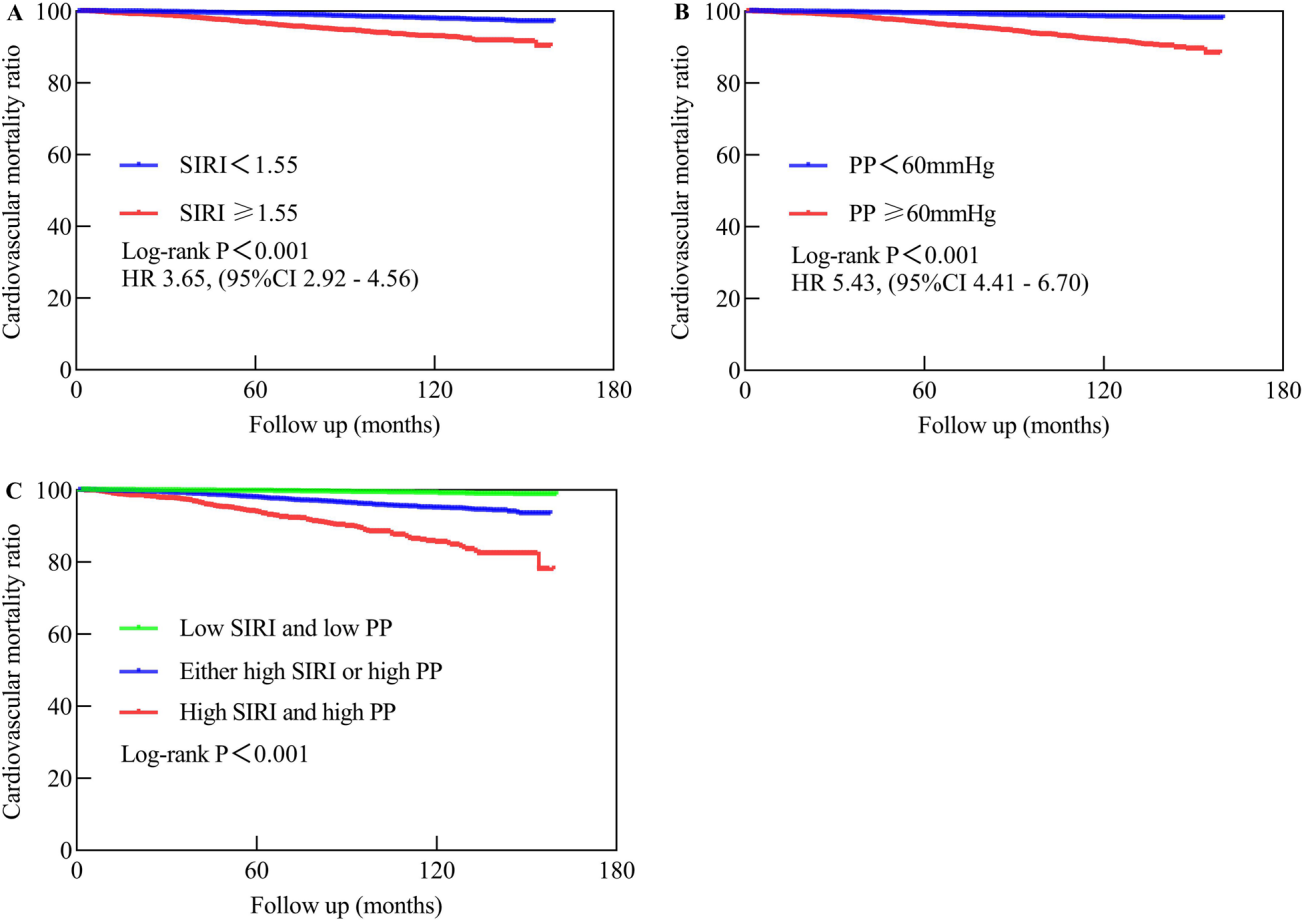
The Kaplan-Meier analysis showed the cardiovascular mortality in different populations. **(A)** Represents low SIRI compared to high SIRI. **(B)** Compares low PP to high PP, while **(C)** compares high SIRI and high PP to either high SIRI or high PP vs. low SIRI and low PP. Low SIRI is defined as an SIRI of less than 1.55, while high SIRI is defined as an SIRI of 1.55 or higher. Low PP is defined as a PP of less than 60 mmHg, while high PP is defined as a PP of 60 mmHg or higher.

Kaplan-Meier survival curves were generated based on cutoff values of SIRI and PP, along with data on CVM. The high SIRI had a higher mortality compared to the low SIRI, with a Log-rank *P* < 0.001 and an HR of 3.65 (95% CI: 2.92–4.56). Similarly, the high PP had a higher mortality compared to the low PP, with a Log-rank *P* < 0.001 and an HR of 5.43 (95% CI: 4.41–6.70). The groups comprising high SIRI and high PP, either high SIRI or high PP, exhibited higher mortality than those of the low SIRI and low PP groups (Log-rank *P* < 0.001), as shown in Figure 4.

### Discrimination and calibration of the nomogram

**Figure 5:**
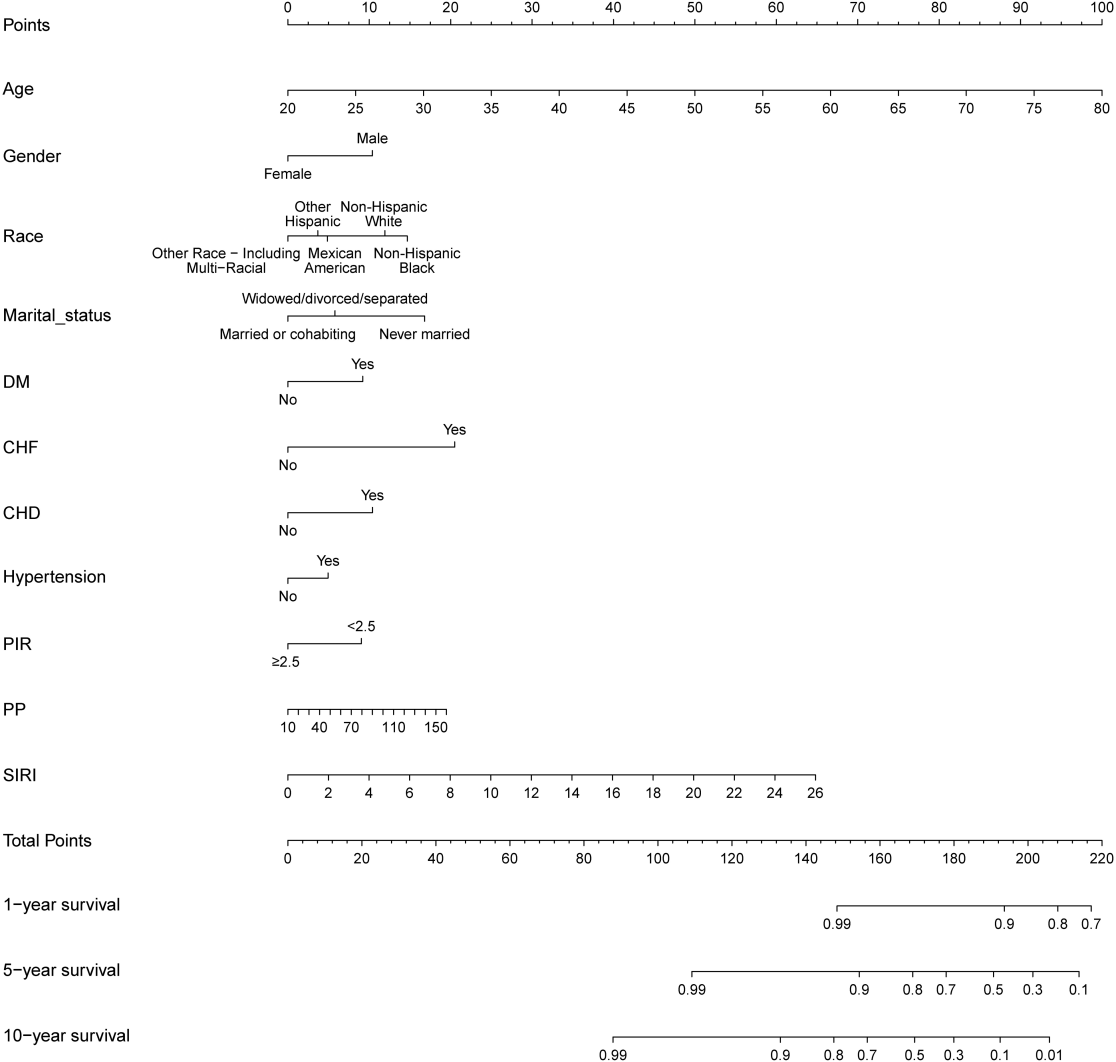
A novel prognostic nomogram for cardiovascular mortality across 1, 5, and 10-year intervals, wherein the cumulative score aligned with the probability of cardiovascular mortality depicted at the base.

**Figure 6:**
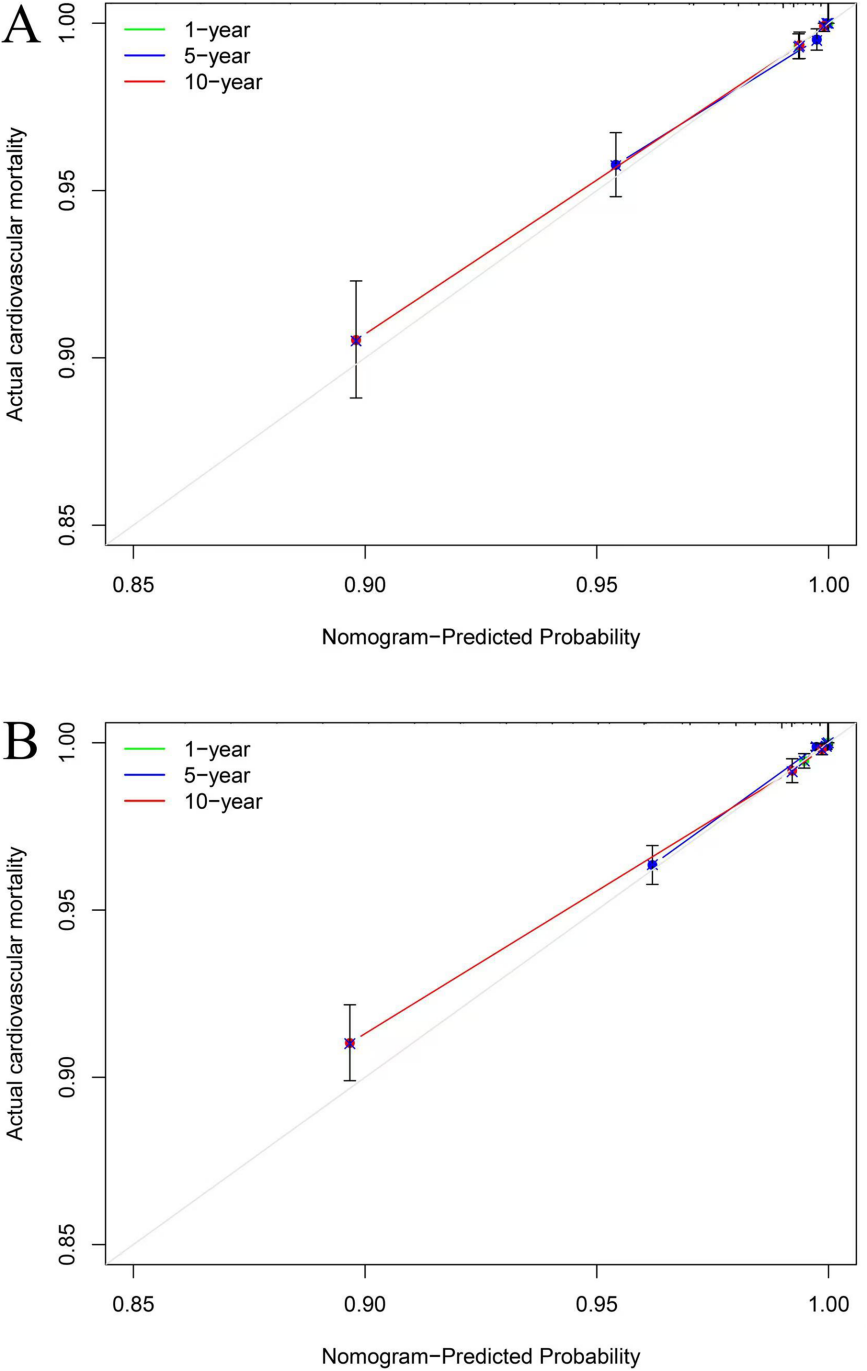
The calibration plots for cardiovascular mortality (CVM). The *x*-axis represents the predicted risk of CVM. The *y*-axis represents the actual rate of CVM. The grey line indicates a perfect prediction by an ideal model. The green, blue, and red solid lines represent the performance of the predicting model, with a closer fit to the grey line suggesting better prediction for 1-year, 5-year, and 10-year CVM. The C-index of the internal validation cohort was 0.90 **(A)**, and the external validation cohort was 0.91 **(B)**.

Our nomogram comprises 11 independent risk factors, including general risk factors, SIRI, and PP (Figure 5). The AUC for the combined general risk factors was 0.88 (95% CI: 0.87–0.90), while the AUC for general risk factors + SIRI + PP was 0.89 (95% CI: 0.87–0.90). Using the *Z*-test to compare general risk factors + SIRI + PP and general risk factors, a statistically notable disparity was detected (Z = 4.17, *P* < 0.001) (Supplementary Figure 4 and Table 2B). We also employed 1,000 bootstrap samples to calculate the C-index for assessing the predictive accuracy of the nomogram, yielding a C-index of 0.90, suggesting excellent predictive value. The C-index of the internal validation cohort was 0.90, and the external validation cohort was 0.91. As depicted in Figure 6, the calibration plot of predicted probabilities demonstrated excellent alignment with the actual CVM rates at 1, 5, and 10 years.

## Discussion

Our research findings suggest that the accuracy of predicting cardiovascular mortality (CVM) may be enhanced through the simultaneous integration of systemic inflammatory response index (SIRI) and pulse pressure (PP). This improvement appears to show a considerable degree of robustness and consistency, particularly in female cohorts, non-smokers, and non-pathological contexts. We observe that higher levels of SIRI and PP are associated with increased mortality. Our study offers preliminary evidence that SIRI may perform better than systemic immune-inflammation index and systemic immune-inflammation response index in predicting CVM within the general population and indicates an S-shaped relationship with PP. Furthermore, we have developed an innovative nomogram to provide precise predictive capabilities for CVM at 1, 5, and 10 years.

Chronic inflammation plays a pivotal role in the pathogenesis of cardiovascular diseases (CVD) and associated mortality, with the SIRI emerging as a significant biomarker reflecting the overall inflammatory state. Elevated SIRI levels have been demonstrated to correlate closely with CVM (2), indicating that persistent inflammation contributes not only to endothelial dysfunction but also to the progression of atherosclerosis (25, 26). Under conditions of chronic inflammation, endothelial cell injury disrupts vascular function and hemodynamics, thereby increasing the risk of cardiovascular events (27).

Concurrently, elevated PP is also significantly linked to the risk of CVM (14). PP serves as an indicator of arterial elasticity and compliance; its elevation is typically associated with arterial stiffness and a decline in vascular compliance (28). Research has established that high PP is not merely a risk factor for cardiovascular diseases and CVM but can exacerbate cardiac workload, leading to alterations in cardiac structure and function (14, 29). Furthermore, PP elevation is significantly associated with various inflammatory markers, such as C-reactive protein and interleukin-6, suggesting that inflammation may exacerbate cardiovascular risk by affecting vascular compliance (30–32).

Importantly, the interrelationship between SIRI and PP highlights their collective capacity to reflect the state of chronic inflammation and vascular health. In our study, when SIRI is greater than 0.51, PP gradually increases with the increase in SIRI, indicating that under inflammatory conditions, vascular functionality may be severely compromised. Specifically, chronic inflammation leads to endothelial activation and the infiltration of inflammatory cells, thereby heightening mechanical stress on the vascular wall and contributing to increased PP (30, 32). Conversely, elevated PP can impose additional tensile stress on the vessel wall, potentially triggering further endothelial damage and inflammatory responses, thus perpetuating a vicious cycle.

In our study, the synergistic application of SIRI and PP demonstrates a stronger predictive efficacy compared to their individual use. Our findings confirm our hypothesis that the simultaneous presence of high SIRI and low PP can further increase the risk of CVM, and this conclusion is consistent across various sensitivity analyses. Therefore, integrating SIRI and PP as predictive markers for CVM holds significant clinical relevance. By assessing these two indices, clinicians can obtain a comprehensive understanding of a patient's cardiovascular risk, facilitating the development of personalized intervention strategies. This combined assessment enhances the predictive accuracy of cardiovascular events but also aids in identifying high-risk individuals, enabling early intervention and improved prognostic outcomes.

Subgroup analysis shows that compared to individuals with lower SIRI and PP levels, individuals in the high SIRI and high PP subgroup have a higher proportion of widowed, divorced, or separated, higher smoking rates, increased comorbidity rates, higher BMI, and lower proportions of individuals with a college education or above. Following adjustments for numerous confounding variables, high SIRI and PP, as well as either high SIRI or PP, were associated with increased CVM compared to low SIRI and PP, with robust results also obtained in sensitivity analyses. Notably, as the levels of SIRI and PP increased, there was a discernible upward trend in the risk of CVM within the wider community, which achieved statistical significance. Furthermore, subgroup analysis shows that the combined use of the SIRI and PP has predictive value (AUC 0.77–0.79) in different populations, particularly evident among female cohorts, non-smokers, and non-pathological contexts. Potential explanations may involve the relatively diminished significance attributed to SIRI and PP concerning mortality among patients burdened with comorbidities. Additionally, given that individuals with comorbidities undergo frequent assessments and hospitalizations, aberrations in SIRI and PP are more readily identifiable, thereby facilitating the management of these parameters within a more favorable range through lifestyle adjustments adherence to standardized medication regimens, and even timely surgical interventions. Lastly, in contrast to males, females demonstrate lower rates of smoking, alcohol consumption and BMI, along with decreased incidences of diabetes mellitus (DM), CHF, CHD, and CVM. Similarly, comorbidities are more prevalent among smokers. Consequently, the relative importance of SIRI and PP in mortality is accentuated among females and non-smokers. This implies that timely identification, diagnosis, and management of abnormal SIRI and PP are imperative in non-pathological conditions and among females. Moreover, the joint utilization of SIRI and PP demonstrates applicability in predicting CVM within the general citizenry.

We know that ischemic heart disease, stroke, self-inflicted injury, chronic obstructive pulmonary disease, and cancer are the main causes of mortality (33). Major risk factors contributing to years of life lost due to disability encompass elevated BMI, smoking, hypertension, high sodium diet, elevated fasting blood glucose, medication usage, and environmental particulate pollution. Considering these risk factors, our nomogram amalgamates general risk factors while integrating two novel risk factors identified in our study, namely SIRI and PP, totaling 11 independent risk factors. Our investigation has unveiled that the incorporation of SIRI and PP into the novel clinical prediction model enhances its prognostic capability, with statistically significant disparities noted. Furthermore, the concordance index of 0.90 indicates commendable discriminative ability, consistent with the cumulative area under the curve calculations and surpassing previous study reports (17–19). Calibration curves evince optimal alignment between predicted and actual CVM rates at 1, 5, and 10 years.

### Strengths and limitations

First, SIRI and PP are easily obtainable indicators in clinical practice, and their combination has a higher predictive value for CVM. Second, multiple sensitivity analyses confirmed that the group with higher SIRI and PP had a higher CVM rate. Finally, the new nomogram model demonstrated a higher c-index, providing valuable guidance in clinical work.

However, notable limitations exist. Primarily, the study encountered challenges related to missing data, potentially introducing bias into the results. Additionally, parameters such as SIRI and PP mainly represent momentary values susceptible to influence by other confounding factors, thus potentially failing to reflect the body's long-term metabolic status. The integration of 24-h dynamic blood pressure monitoring may bolster the robustness of the study's findings. Furthermore, despite the favorable predictive value of the combined SIRI and PP, their sensitivity and specificity remain relatively modest. Lastly, future research could further explore the shared pathways between inflammatory and hypertension pathways. This will help guide subsequent studies in this field.

## Conclusions

The concurrent utilization of SIRI and PP emerges as a robust predictor of CVM within the general populace of the US. Particular attention to assessment and management may be warranted in female cohorts, non-smokers, and non-pathological contexts. Both SIRI and PP are autonomous risk factors for CVM in the broader demographic, with elevated levels of both indicators correlating with heightened risks of CVM. The novel nomogram exhibits relatively high precision in forecasting CVM.

## Data Availability

The datasets presented in this study can be found in online repositories. The names of the repository/repositories and accession number(s) can be found in the article/Supplementary Material.

## Supplementary material

Supplementary Figure 1 The flowchart of the study.

Supplementary Figure 2 Incorporating indicators like SIRI, SII, and SIIRI to assess cardiovascular mortality, resulted in AUC values of 0.67, 0.60, and 0.62, respectively.

Supplementary Figure 3 Incorporating indicators like PP, SBP, DBP, and MAP to assess cardiovascular mortality, resulted in AUC values of 0.73, 0.67, 0.61, and 0.52, respectively.

Supplementary Figure 4 The cumulative AUCs for cardiovascular mortality for general risk factors and general risk factors + PP + SIRI were 0.85 and 0.86, respectively.
