# Supplementary figures and images for: Integration of the systemic inflammatory response index with pulse pressure enhances prognostication of cardiovascular mortality in the general population of the United States: insights from the NHANES database
Source: Front Cardiovasc Med. 2024 Nov 18;11:1439239. doi: 10.3389/fcvm.2024.1439239 (PMC11609212; doi:10.3389/fcvm.2024.1439239)

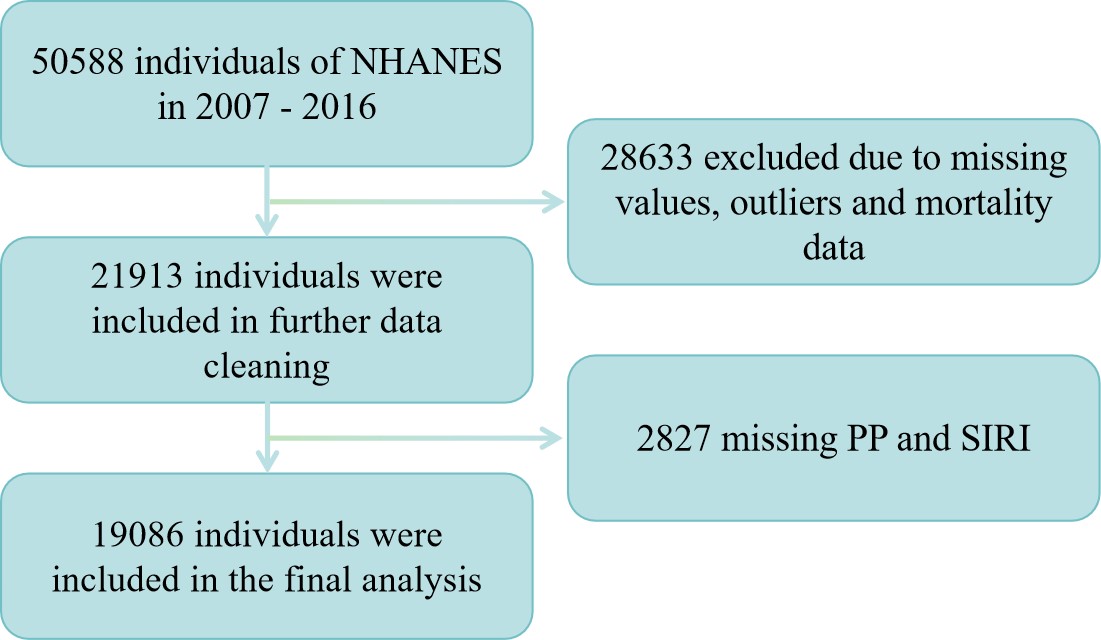

Supplement: Supplementary Figure 1 — The flowchart of the study. [file Image1.jpeg]
